# Chitosan-Grafted Halloysite Nanotubes-Fe_3_O_4_ Composite for Laccase-Immobilization and Sulfamethoxazole-Degradation

**DOI:** 10.3390/polym12102221

**Published:** 2020-09-27

**Authors:** Avinash A. Kadam, Surendra K. Shinde, Gajanan S. Ghodake, Ganesh D. Saratale, Rijuta G. Saratale, Bharat Sharma, Seunghun Hyun, Jung-Suk Sung

**Affiliations:** 1 Research Institute of Biotechnology and Medical Converged Science, Dongguk University-Seoul, Ilsandong-gu, Goyang-si, Gyeonggi-do 10326, Korea; kadamavinash@dongguk.edu (A.A.K.); rijutaganesh@gmail.com (R.G.S.); 2 Department of Biological and Environmental Science, Dongguk University-Seoul, Ilsandong-gu, Goyang-si, Gyonggido 10326, Korea; surendrashinde.phy@gmail.com (S.K.S.); ghodakegs@gmail.com (G.S.G.); 3 Department of Food Science and Biotechnology, Dongguk University-Seoul, Ilsandong-gu, Goyang-si, Gyeonggido 10326, Korea; gdsaratale@dongguk.edu; 4 Department of Materials Science and Engineering, Incheon National University, Academy Road Yeonsu, Incheon 22012, Korea; bharatsharma796@gmail.com; 5 Department of Environmental Science and Ecological Engineering, Korea University, Seoul 02841, Korea; soilhyun@korea.ac.kr; 6 Department of Life Science, College of Life Science and Biotechnology, Dongguk University-Seoul, Ilsandong-gu, Goyang-si, Gyonggido 10326, Korea

**Keywords:** chitosan, laccase-immobilization, nano-engineered supports, super-magnetic separation, sulfamethoxazole degradation

## Abstract

A surface-engineered nano-support for enzyme laccase-immobilization was designed by grafting the surface of halloysite nanotubes (HNTs) with Fe_3_O_4_ nanoparticles and chitosan. Herein, HNTs were magnetized (HNTs-M) by a cost-effective reduction-precipitation method. The synthesized HNTs-M were grafted with 0.25%, 0.5%, 1%, and 2% chitosan (HNTs-M-chitosan), respectively. Synthesized HNTs-M-chitosan (0.25%), HNTs-M-chitosan (0.5%), HNTs-M-chitosan (1%) and HNTs-M-chitosan (2%) were linked with glutaraldehyde (GTA) for laccase immobilization. Among these formulations, HNTs-M-chitosan (1%) exhibited the highest laccase immobilization with 95.13% activity recovery and 100.12 mg/g of laccase loading. The optimized material was characterized thoroughly by scanning electron microscopy (SEM), high-resolution transmission electron microscopy (HR-TEM), X-ray powder diffraction (XRD), thermal gravimetric analysis (TGA), and vibrating sample magnetometer (VSM) analysis. The immobilized laccase (HNTs-M-chitosan (1%)-GTA-*Laccase*) exhibited higher pH, temperature, and storage stabilities. The HNTs-M-chitosan (1%)-GTA-*Laccase* possesses excellent reusability capabilities. At the end of 10 cycles of the reusability experiment, HNTs-M-chitosan (1%)-GTA-*Laccase* retained 59.88% of its initial activity. The immobilized laccase was utilized for redox-mediated degradation of sulfamethoxazole (SMX), resulting in 41%, 59%, and 62% degradation of SMX in the presence of 2,2′-Azino-bis(3-ethylbenzothiazoline-6-sulfonic acid) diammonium salt (ABTS), guaiacol (GUA), and syringaldehyde (SA), respectively. Repeated SMX degradation (57.10% after the sixth cycle) confirmed the potential of HNTs-M-chitosan (1%)-GTA-*Laccase* for environmental pollutant degradation. Thus, we successfully designed chitosan-based, rapidly separable super-magnetic nanotubes for efficacious enhancement of laccase biocatalysis, which can be applied as nano-supports for other enzymes.

## 1. Introduction

Laccase is a multi-copper oxidoreductase, which catalyzes single-electron substrate oxidation using molecular oxygen [1]. Biocatalyst enzymes have long been explored, due to their high specificity, catalytic power, and stereo-selectivity [2]. Enzyme immobilization processes gave us bio-based catalysts with enhanced properties for diverse applications [3,4]. Laccase is a capable biocatalyst for micro-pollutant removal [5]. Enormous population growth has increased the industrial agricultural demands, resulting in increasing micro-pollutant concentrations in the environment. The increased concentration of micro-pollutants in the environment due to constant release, even at trace concentrations (ng/L), from wastewater-treatment plants into water bodies is of great environmental and public health concern [6]. Hence, in this study, the pharmaceutical compound and indicator of antibiotic pollution ‘sulfamethoxazole (SMX) was chosen as the target pollutant for immobilized-laccase-based bio-catalytical degradation. SMX is known for its widespread use in human and veterinary medicines, a frequent occurrence in water supplies, and resultant negative health consequences. [7].

The development of efficient nano-supports is required to use laccase for the desired environmental application [5]. There is a huge opportunity in the development of an excellent nano-supports, which can be used in enzyme-based catalysis [2]. Among various nano-supports, halloysite nanotubes (HNTs) have recently attracted significant attention [8], due to the excellent physical properties of HNTs, including their highly-modifiable surfaces, nano-tubular morphology, the nano-lumen inside the nanotube, interior and exterior modifiable surfaces, their robust nature, high surface-area (up to 184.9 m^2^/g), and large pore volume (up to 0.353 cm^3^/g) [9]. Due to these properties, HNTs are used as effective carriers for enzyme immobilization [10], drug delivery [11], adsorbents [12], photo-catalysis [13], and other applications. 

However, there is still a major opportunity to further modify the HNT surface [14]. To develop HNTs as a nano-support for laccase immobilization, it is crucial to incorporate magnetic properties because magnetism allows for rapid separation from water [15]. There are many approaches available for the incorporation of magnetic nanoparticles in HNTs; however, the reduction-precipitation method is cost-effective, requires ambient temperatures, and does not utilize additional N_2_ gas [16]. Hence, this study focuses on the reduction-precipitation based magnetization of HNTs. Further, functionalization of the HNTs was required for the immobilization process. Chitosan is a natural cationic polymer composed of β-1-4 linked D-glucosamine (deacetylated unit) and N-acetyl-D-glucosamine (acetylated unit) [17]. In last two decades, chitosan emerged as a highly important biopolymer that can be utilized for diverse applications [18]. Chitosan and its hybrid with other organic-inorganic materials in the form of nano/micro formulations is attracting the attention in the applications such as biomedical technology, remediation, catalysis, food packaging, and cultural heritage treatment etc. [19,20]. Polysaccharides/HNTs hybrids as smart bio-nano-composite materials for diverse applications have been reviewed by Bertolino et al., 2020 [21]. Chitosan possesses ubiquitous (–NH_2_) functional groups. Given its natural origin, chitosan-modified HNTs have several advantages over the chemically (–NH_2_) modified HNTs [10]. However, an optimization of the amount of chitosan onto the HNT surface for enhanced laccase immobilization and degradation of SMX has not yet been reported [22]. 

In this work, we developed a reduction-precipitation based procedure for the magnetization of HNTs, optimized the chitosan loading on the magnetized HNT surface for enhanced laccase immobilization, studied the biocatalytic properties of the immobilized laccase, and assessed the ability of immobilized laccase to degrade SMX in water. As a result, we developed a chitosan-based, efficient nano-support that can be applied for enzyme immobilization and environmental pollutant decontamination. 

## 2. Materials and Methods

### 2.1. Materials

Halloysite nanoclay (diameter × length- 30–70 nm × 1–3 μm, nanotube), sulfamethoxazole (SMX, analytical standard), laccase from *Trametes versicolor* (form- powder), sodium sulfite (Na_2_SO_3_, ACS reagent grade), sodium hydroxide (NaOH, ACS reagent grade), 2,2′-Azino-bis(3-ethylbenzothiazoline-6-sulfonic acid) diammonium salt (ABTS, assay- ≥98% high-performance liquid chromatography (HPLC)), syringaldehyde (SA, assay- ≥98%), guaiacol (GUA, assay- ≥98%), methanol (HPLC grade), glutaraldehyde (GTA, grade II, 25% in H_2_O), and chitosan (75–85% deacetylated, low molecular weight, molecular weight- 50,000–190,000 Da) were obtained from Sigma-Aldrich (St. Louis, MO, USA). FeCl_3_-6H_2_O was obtained from JUNSEI (Kyoto) Japan. Laccase from *Trametes versicolor* have a molecular mass of 70 kDa and an isoelectric point (pI) of 3.5 [23].

### 2.2. Synthesis of HNTs-M-Chitosan

The HNTs-M-chitosan was synthesized in two steps. First, pristine HNTs were modified with Fe_3_O_4_ NPs [16]. Then, Fe_3_O_4_ NPs-modified HNTs were functionalized with the chitosan. In the first step, 0.5–2 g of HNTs were added to 100 mL of deionized water. The mixture was ultrasonicated by Sonics Vibra-Cell VC130 Ultrasonic Processor, power—130 W, frequency—20 kHz, and amplitude—60 µM (Sonics & Materials, Inc., Newtown, CT, USA) for 1 h. The mixture was then added to the 50 mL of 3% FeCl_3_-6H_2_O solution and rapidly vortexed. Then, 0.7% of Na_2_SO_3_ solution was added. The addition of Na_2_SO_3_ solution turns the solution color red. Once the red color returned to yellow, 30 mL of 1 N NaOH was added. This turns the mixture black due to the colored precipitate of Fe_3_O_4_ NPs-modified HNTs. The black-colored composites were magnetically recovered and washed thoroughly with distilled water, ethanol, and methanol. The obtained sample was dried in an oven at 60 °C for 48 h. The obtained sample of HNTs-M was powdered stored for further use. The chitosan modification of HNTs-M was done in a 500 mL beaker. The chitosan solutions (0.25%, 0.5%, 1% and 2%) in 50 mL of 2% acetic acid were vortexed for 4 h. The clear 0.25%, 0.5%, 1%, and 2% chitosan solutions were added to 0.5 g of HNTs-M in 50 mL distilled water and ultrasonicated Sonics Vibra-Cell VC130 Ultrasonic Processor, power—130 W, frequency—20 kHz, and amplitude—60 µM (Sonics & Materials, Inc., Newtown, CT, USA) for 15 min. The resulting mixtures were refluxed rapidly for 15 min. Then, 2 mL of 2.5% glutaraldehyde was added to the mixtures. After glutaraldehyde addition, the mixtures continued refluxing for 8 h at 50 °C. The obtained mixtures of HNTs-M-chitosan (0.25%), HNTs-M-chitosan (0.5%), HNTs-M-chitosan (1%) and HNTs-M-chitosan (2%) were separated magnetically and washed thoroughly with distilled water. The materials were dried in an oven at 60 °C and powdered for further use in enzyme immobilizations.

### 2.3. Laccase Immobilization on Synthesized Materials

HNTs-M-chitosan (0.25%), HNTs-M-chitosan (0.5%), HNTs-M-chitosan (1%), and HNTs-M-chitosan (2%) were cross-linked with glutaraldehyde (GTA). A total of 100 mg of materials were added to 10 mL of 2.5% GTA solution. The solution was rapidly shaken at 200 rpm for 24 h at 25 °C. The GTA linked materials were separated using a magnet and thoroughly washed with distilled water. The GTA-linked materials HNTs-M-chitosan (0.25%), HNTs-M-chitosan (5%), HNTs-M-chitosan (1%), and HNTs-M-chitosan (2%) were then added to a laccase solution (1 mg/mL) in sodium acetate buffer (100 mM, pH 4). The mixtures were continuously shaken at 200 rpm for 24 h at 20 °C. Afterward, immobilized laccase were separated magnetically and thoroughly washed with the sodium acetate buffer (100 mM, pH 4). The immobilized laccase was immediately tested for activity recovery (%) and laccase loading. The activity recovery (%) was calculated by Equation (1):

(1) $$Activity\;recovery\;\left(\%\right)=A_{I}/A_{F}\times 100$$

where *A_I_* is the activity of the immobilized laccase, and *A_F_* is the initial free laccase activity. The laccase activity was tested in a reaction mixture containing sodium acetate buffer (100 mM, pH 4), ABTS (90 µM) and laccase/immobilized laccase. The laccase/immobilized laccase activity corresponds to the ABTS oxidation in the reaction mixture. The ABTS oxidation was measured by spectrophotometrically at 420 nm [24]. Further, to determine the laccase loading, the laccase concentration in solutions before and after immobilization were tested for protein content using the Bradford method (Pierce™ Coomassie (Bradford) Protein Assay Kit, Thermo Scientific™, Massachusetts, MA, USA) [25]. The Pierce Coomassie Protein Assay Kit is follows the principles originally described by Bradford in 1976. In the acidic condition when proteins mixed with Coomassie-dye reagent, it changes color from brown to blue as per the amount to the amount of protein present. Protein estimation was done by comparison to the color response of protein assay standards. The laccase loading (mg/g) on nanocomposites was calculated by Equation (2):

(2) $$Laccase\;loading\;(\mathrm{mg}/g)=\left(C_{i}-C_{r}\right)V/W$$

where *C_i_* is the initial laccase concentration before immobilization (mg/L), *C_r_* is the retained laccase concentration after immobilization (mg/L), *V* is the volume of the solution in liters (L), and *W* is the weight of the nanocomposites in gram (*g*). The synthesized material with higher laccase loading and activity recovery (%) capacity was further characterized in detail and studied for laccase biocatalysis.

### 2.4. Characterizations of Nano-Supports 

Morphological analysis was performed by scanning electron microscopy (SEM, FC-SM10, Hitachi S-4800, Ibaraki, Japan) and high-resolution transmission electron microscopy (HR-TEM, Tecnai G2 transmission electron microscope, Hillsboro, OR, USA). The crystallinity of the materials was determined using an X-ray powder diffractometer with Cu-Kα radiation (λ= 1.5418 Å) (Ultima IV/Rigaku, Tokyo, Japan). The vibrating sample magnetometer (VSM) analysis was done by Lakeshore, Model: 7407, LA, USA. Thermogravimetric analysis was performed to measure the weight (%) loss with respect to temperature from 25 to 800 °C (TA Instrument, Q600, New Castle, DE, USA). Surface charge of the materials was determined by zeta potential measurements (Zeta-potential and Particle Size Analyzer ELSZ-2000 series, Otsuka Electronics., Co., Ltd. Osaka, Japan)

### 2.5. Immobilized Laccase Biocatalysis 

As mentioned previously, the HNTs-M-chitosan (1%)-GTA-*Laccase* was selected for biocatalysis studies. The laccase loading pattern on HNTs-M-chitosan (1%) was assessed by varying the initial concentration of laccase (0.2 mg/mL, 0.4 mg/mL, 0.6 mg/mL, 0.8 mg/mL, 1 mg/mL, and 1.2 mg/mL). The laccase loading was measured as previously mentioned in Equation (2) from Section 2.3. HNTs-M-chitosan (1%)-GTA-*Laccase* were then tested for thermal, pH, and time stabilities. Thermal stability was studied by incubating free laccase and HNTs-M-chitosan (1%)-GTA-*Laccase* at 60 °C for 220 min. The samples were tested every 40 min to determine relative activity (%). The time stabilities were investigated by incubating free laccase and HNTs-M-chitosan (1%)-GTA-*Laccase* for 30 d at 4 °C. The samples were withdrawn at every 5 d, and tested to determine relative activity (%). The pH stability study was performed by incubating 0.1 mL of free and immobilized laccase in 1 mL of different pH buffers (1–9) for 1 h at 25 °C. For this experiment 100 mM HCl–KCl buffer (for pH 1 and 2), 100 mM citrate–phosphate buffer (for pH 3, 4, 5, 6, and 7), and 100 mM Tris-HCl buffer (for pH 8 and 9) were used. After 1 h of incubation in varying pH conditions, the samples were tested to determine relative activity (%) to measure the respective pH stabilities. Furthermore, the HNTs-M-chitosan (1%)-GTA-*Laccase* nanocomposites were used in repeated cycle studies. The HNTs-M-chitosan (1%)-GTA-*Laccase* was tested for laccase activity in the 1st cycle as described above. Before the 2nd cycle, the HNTs-M-chitosan (1%)-GTA-*Laccase* used in the 1st cycle were washed with sodium acetate buffer (pH 4.0) three times. The washed HNTs-M-chitosan (1%)-GTA-*Laccase* were added to fresh reactants and the laccase activity was measured. A total of 10 of these cycles were performed to assess the potency of the HNTs-M-chitosan (1%)-GTA-*Laccase*. The relative activity is measured as considering the original activity before these experiments as 100%. The percent relative activity is given as Equation (3):

(3) $$Relative\;activity\;\left(\%\right)=A_{a}/A_{o}\times 100$$

where *A_a_* is the activity after the experiment, and *A_o_* is the original activity before experiment. 

### 2.6. SMX Degradation by HNTs-M-Chitosan (1%)-GTA-Laccase

The SMX degradation was carried out by HNTs-M-chitosan (1%)-GTA-*Laccase*. The reaction mixture was as follows: 25 ppm SMX, 1 mM redox mediator (either ABTS, SA or GUA), HNTs-M-chitosan (1%)-GTA-*Laccase* in sodium acetate buffer, pH 4.0. The reaction was carried out with each redox mediator separately for 4 h at 200 rpm and 20 °C. The degradation of the SMX was measured with high-performance liquid chromatography (HPLC, Shimadzu LC-20AD, Kyoto, Japan) at a detection wavelength of 286 nm with a mobile phase of methanol:water (60:40) at a flow rate of 0.6 mL/min with the C18 column (Ascentis^®^ Express C18, Sigma-Aldrich, St. Louis, MO, USA, 2.7 μm HPLC Column 2.7 μm particle size, length × inner diameter- 5 cm × 4.6 mm). The SMX degradation was measured as Equation (4):

(4) $$SMX\;degradation\;\left(\%\right)=P_{std}-P_{deg}/P_{std}\times 100$$

where *P_std_* is peak area of the standard SMX peak before the degradation experiment, and *P_deg_* is peak area of the SMX peak after the degradation experiment. The SMX degradation was also carried out in repeated cycles. After completion of the first cycle, the HNTs-M-chitosan (1%)-GTA-*Laccase* was washed thoroughly with sodium acetate reaction buffer three times. The washed HNTs-M-chitosan (1%)-GTA-*Laccase* was added to fresh reactants for the SMX degradation experiment for 6 cycles.

## 3. Results and Discussion 

### 3.1. Synthesis

**Figure 1:**
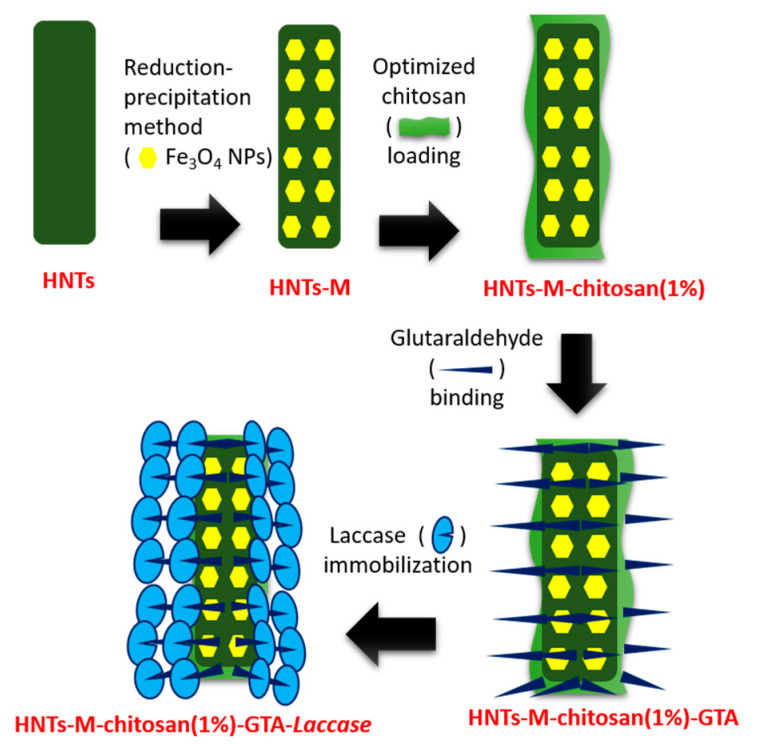
Stepwise synthesis of the halloysite nanotubes (HNTs)-magnetized(M)-chitosan (1%)- glutaraldehyde (GTA)-*Laccase*.

A schematic representation of the stepwise synthesis of HNTs-M-chitosan (1%)-GTA-*Laccase* is shown in Figure 1. Pristine HNTs were first super-magnetized with the Fe_3_O_4_ NPs. This was done by the typical reduction precipitation method. In the reaction mechanism, the Fe^3+^ ions first attach to the surface of pristine HNTs. Further addition of Na_2_SO_3_ partially reduces the attached Fe^3+^ ions to Fe^2+^. At the same time, addition of NaOH synthesizes Fe_3_O_4_ NPs on the surface of HNTs (HNTs-M). The reduction precipitation is a highly cost-effective, easy, and reliable method compared to the typically-used co-precipitation method [22]. 

It is crucial to separate the nano-supports loaded with enzyme efficaciously from solution; this simple magnetization boosts the separation potential of HNTs-M-chitosan-GTA. After magnetization, the HNTs-M surface was decorated with varying chitosan concentrations. The chitosan-modified HNTs-M (HNTs-M-chitosan) were further modified by the cross-linker GTA (HNTs-M-chitosan-GTA). The unique characteristics of GTA render it one of the most effective protein crosslinking reagents [26]. The GTA cross-links the laccase onto the HNTs-M-chitosan. The laccase-loaded HNTs-M-chitosan was further assessed for immobilization parameters and the degradation of SMX.

### 3.2. Laccase Immobilization on Developed Nano-Supports

**Figure 2:**
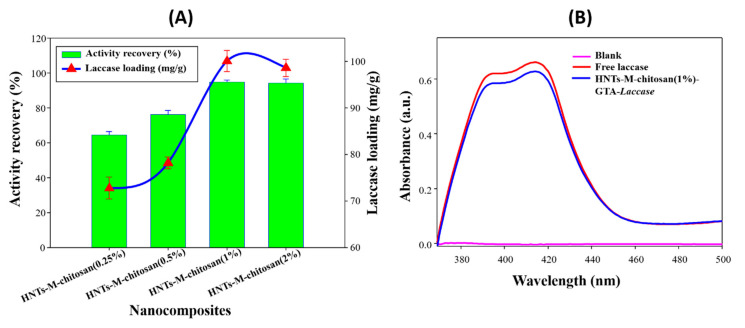
(**A**) Activity recovery (%) and laccase loading (mg/g) of HNTs-M-chitosan (0.25%), HNTs-M-chitosan (0.5%), HNTs-M-chitosan (1%) and HNTs-M-chitosan (2%); and (**B**) 2,2′-Azino-bis(3-ethylbenzothiazoline-6-sulfonic acid) diammonium salt (ABTS) oxidation spectra of blank (only ABTS), oxidized ABTS after 5 min by free laccase, and oxidized ABTS after 5 min by HNTs-M-chitosan (1%) at pH 4, temperature 25 °C.

Varying chitosan concentrations (0.25% to 2%) were loaded onto the HNTs-M surface. Chitosan served as the main source of (-NH_2_) functional groups required for enzyme immobilization in this process. Thus, optimizing the chitosan concentration on the HNTs-M surface is crucial. However previous reports on chitosan-modified HNTs do not explore this parameter [16,27]. Figure 2A shows the activity recovery (%) and laccase loading (mg/g) potential of HNTs-M-chitosan (0.25%), HNTs-M-chitosan (0.5%), HNTs-M-chitosan (1%) and HNTs-M-chitosan (2%). These results show that increasing the chitosan concentration on the HNTs surface enhances laccase immobilization (Figure 2A). The laccase loading potential of the nanocomposites are very important to know its enzyme immobilization efficiency. HNTs-M-chitosan (0.25%), HNTs-M-chitosan (0.5%), HNTs-M-chitosan (1%), and HNTs-M-chitosan (2%) exhibited 64%, 76%, 95%, and 94% activity recoveries, respectively, and 72 mg/g, 78 mg/g, 100 mg/g, and 98 mg/g of laccase loading, respectively. Thus, HNTs-M-chitosan (1%) performed better than other materials studied HNTs-M-chitosan (0.25%), HNTs-M-chitosan (0.5%), and HNTs-M-chitosan (2%) (Figure 2A). With increase in chitosan concentration from 0.25% to 1%, the immobilization performance increased sequentially. The HNTs-M-chitosan (1%) exhibited the best laccase immobilization. This might be due to the optimum chitosan (-HN_2_) functional groups attained at chitosan mass of 1%. Further increase in the chitosan concentration might caused the overloading of chitosan (-HN_2_) functional groups, and resulted in decrease of laccase loading in HNTs-M-chitosan (2%). The details of the recent nano-supports for laccase loading capacities were mentioned in the Table 1. It showed that the obtained laccase loading capacity of HNTs-M-chitosan (1%) is quite higher than recently reported materials for laccase-immobilization in Table 1.

In addition, HNTs-M-chitosan (1%) in solution have good colloidal stability. Since potential applications take place in dispersions, particle aggregation should be prevented, in order provide homogeneously distributed primary particles of high surface area [35,36,37]. Moreover, Figure 2B presents the ABTS oxidation spectra by free laccase and immobilized laccase on HNTs-M-chitosan (1%). The excellent retention of the activity by immobilized laccase can be clearly seen in the obtained spectra’s (Figure 2B). This is due to the optimized chitosan loading onto the HNTs-M surface; the laccase immobilization plateaued at the 2% chitosan concentration, likely due to saturation of chitosan on the HNTs-M surface. Hence, HNTs-M-chitosan (1%) was further characterized to study its immobilized laccase bio-catalytical properties.

### 3.3. Characterizations

**Figure 3:**
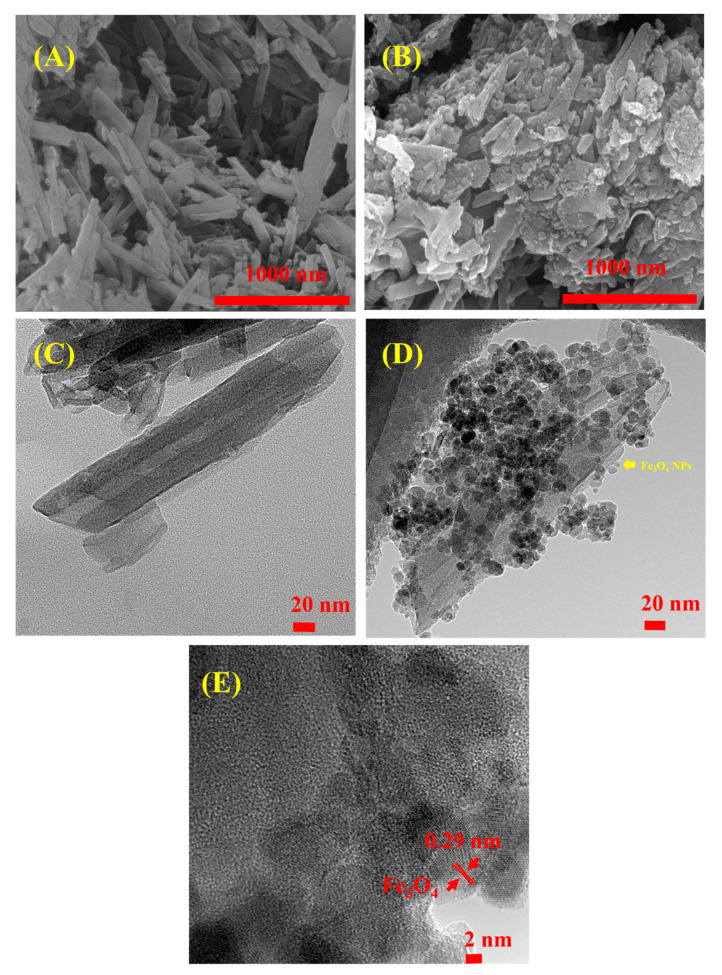
Scanning electron microscopy (SEM) analysis of (**A**) HNTs; and (**B**) HNTs-M-chitosan (1%). high-resolution transmission electron microscopy (HR-TEM) analysis of (**C**) HNTs; (**D**) HNTs-M-chitosan (1%); and (**E**) Zoomed surface of HNTs-M-chitosan (1%), showing the lattice fringe regions of Fe_3_O_4_ NPs loaded over HNTs.

The morphology of the pristine HNTs and HNTs-M-chitosan (1%) was observed using SEM and HR-TEM (Figure 3). Figure 3A shows bare HNTs under SEM. The surface of the tubes can be easily observed. The surface of the tubes appears unmodified, planar, and nanotubular in nature. Similar observations were noted in previous work [14]. There are different lengths and widths of the nanotubes in Figure 3A. Figure 3B shows the HNTs-M-chitosan (1%) morphology. The surface of the HNTs was clearly modified with Fe_3_O_4_ NPs (Figure 3B). The nanotubes were broadened due to the chitosan loading. Further characterization was performed with HR-TEM (Figure 3C–E). Figure 3C shows the pristine HNTs. The inner lumen is clearly evident. The surface of HNTs is unmodified. However, the HNTs-M-chitosan (1%) surface appears intensively modified with Fe_3_O_4_ NPs and chitosan. The broadened, layered morphology of the tubes may be due to the chitosan modification (Figure 3D). Moreover, the lattice fringe region of Fe_3_O_4_ NPs on HNTs was determined to be 0.29 nm (Figure 3E), similar to that seen in prior work [38]. Thus, SEM and HR-TEM images confirmed the nanotubular morphology with desired surface modifications.

**Figure 4:**
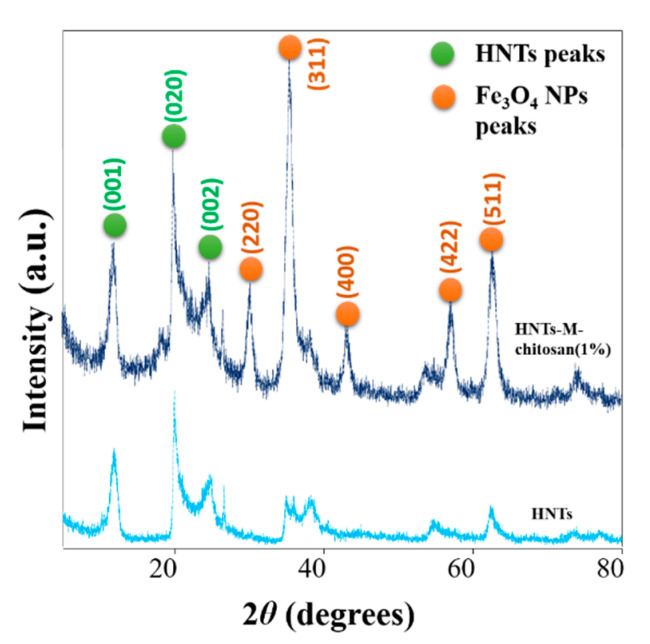
XRD analysis of HNTs and HNTs-M-chitosan (1%).

The crystallinity analysis was performed by determining the X-ray powder diffraction (XRD) patterns of pristine HNTs and HNTs-M-chitosan (1%) (Figure 4). The HNTs and HNTs-M-chitosan (1%) showed typical XRD peaks 11.82°, 19.94°, and 24.55°, which mainly corresponds to HNTs crystalline planes of (001), (020) and (002), respectively (JCPDS 29-1487) [27]. The sharp peak at (2θ = 11.82°) in HNTs-M-chitosan (1%) indicates basal space reflections of the nanotubular multiwall structure of HNTs [27]. This suggest the HNTs nanotubular nature was consistent after the surface modifications. However, in HNTs-M-chitosan (1%), additional peaks of Fe_3_O_4_ NPs 30.22°, 35.37°, 43.23°, 57.13°, and 62.68°, which corresponds to their respective indices (220), (311), (400), (422), and (511), respectively, was observed (JCPDS 19-0629) [39]. This result confirmed the Fe_3_O_4_ NPs modified the HNTs surface. The HNTs and the Fe_3_O_4_ NPs diffraction peaks are marked with light green circles and orange circles, respectively (Figure 4). Thus, the chitosan and Fe_3_O_4_ NP modifications do not alter the basic crystalline nanotubular structure of HNTs. The XRD results were in strong agreement with the morphological observations made by the SEM and HR-TEM analyses (Figure 3). 

**Figure 5:**
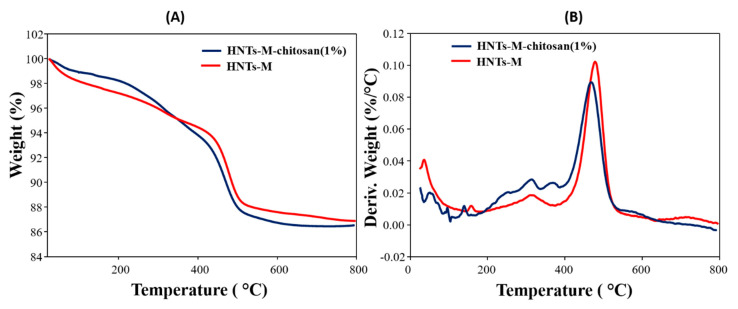
TGA (**A**) and derivative thermogravimetry (DTG); (**B**) analysis of HNTs-M and HNTs-M-chitosan (1%).

The TGA and DTG (derivative thermogravimetry) data for HNTs-M and HNTs-M-chitosan (1%) are shown in Figure 5A,B. The differences in the thermal degradation patterns of the two samples are clearly seen. The initial weight loss from 20–120 °C is associated with the loss of adsorbed water in both samples. Further, the thermal degradation from 353 to 750 °C depicted higher weight loss % in HNTs-M-chitosan (1%) than HNTs-M. At the 700 °C, weight loss exhibited by HNTs-M and HNTs-M-chitosan (1%) were 12.8% and 13.52%, respectively (Figure 5A). The increase in weight loss % in HNTs-M-chitosan (1%) suggest the surface modification of the HNTs-M with chitosan. To get more clear idea regarding thermal degradation patterns, DTG analysis was done (Figure 5B). DTG analysis of HNTs-M-chitosan (1%) showed peaks at 52, 94, 138, 252, 314 and 369 °C, and HNTs-M exhibited peaks at 37, 157 and 315 °C (Figure 5B). The peaks observed in the range of 30 to 200 °C associated with the loss of moisture and surface bound water. The additional peaks of 252 and 369 °C in HNTs-M-chitosan (1%) were demonstrated the thermal degradation of chitosan bound to the surface of HNTs-M. Similar patterns were found for HNTs-M loaded with N-cyclohexyl-2-benzothiazole sulfenamide (CZ) [40]. 

**Figure 6:**
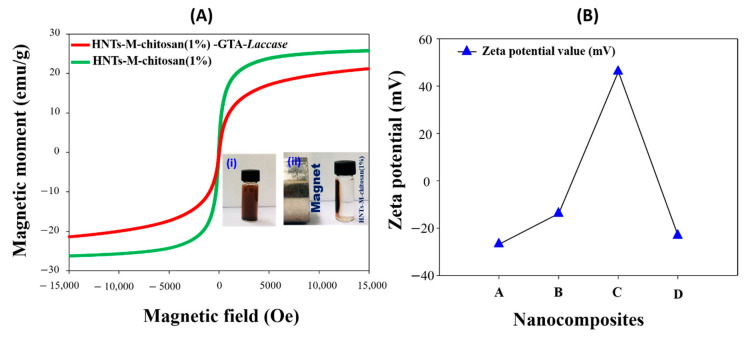
(**A**) Vibrating sample magnetometer (VSM) analysis of HNTs-M-chitosan (1%) and HNTs-M-chitosan (1%)-GTA-*Laccase* (i) HNTs-M-chitosan (1%) without magnet and (ii) HNTs-M-chitosan (1%) with magnet; and (**B**) zeta potential analysis of A- HNTs, B- HNTs-M, C- HNTs-M-chitosan (1%) and D- HNTs-M-chitosan (1%)-GTA-*Laccase* in the water solution at temperature of 25 °C.

It is crucial to measure the magnetic separation potential of the nanocomposite before and after laccase immobilization. Figure 6A shows the VSM analysis of HNTs-M-chitosan (1%) and HNTs-M-chitosan (1%)-GTA-*Laccase*. After laccase loading, the magnetization was decreased, corroborating the successful loading of the laccase onto the HNTs-M-chitosan (1%) nanotubes. The magnetic saturation values found to be 25 emu/g and 21 emu/g for HNTs-M-chitosan (1%) and HNTs-M-chitosan (1%)-GTA-*Laccase*. The hysteresis curves confirmed the super-para-magnetic nature of both materials [10]. The surface charge of the stepwise modified materials was measured with the zeta potential analysis. The detailed results given in the Figure 6B. The obtained results suggest that HNTs, HNTs-M, HNTs-M-chitosan (1%), and HNTs-M-chitosan (1%)-GTA-*Laccase* have zeta potential of −26.65, −13.72, 46.15, and −23.00, respectively. The HNTs possessed negative change on its surface (zeta potential −26.65). The Fe_3_O_4_ modification of HNTs reduced its surface negative charge (zeta potential −13.72). Further, chitosan modification of HNTs-M gave positive surface (zeta potential 46.14). This is the result of anionic polymer chitosan coating over the HNTs-M. Finally, the laccase loaded HNTs-M-chitosan (1%) gave the negative value (zeta potential-23.00). The obtained zeta potential values clearly evidenced the surface charge variation with subsequent surface modifications made on HNTs.

### 3.4. Bio-Catalytic Properties of HNTs-M-Chitosan (1%)-GTA-Laccase

**Figure 7:**
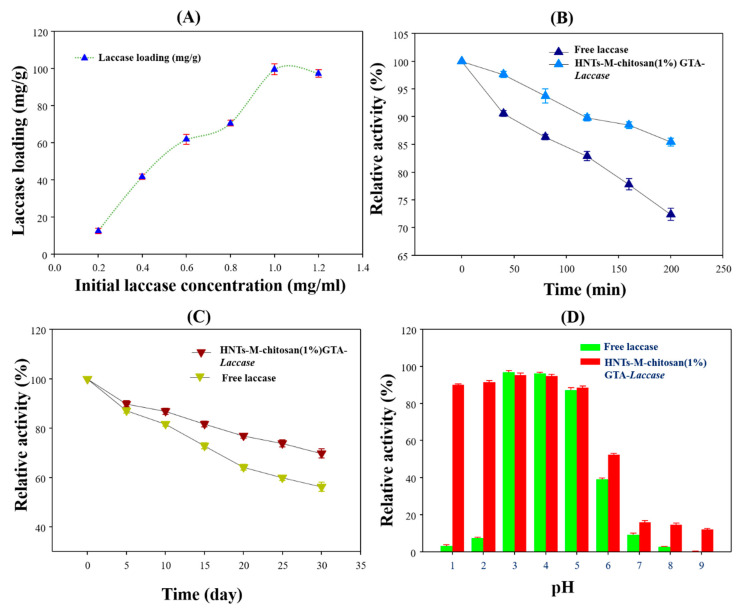
(**A**) Effect of initial laccase concentration on its loading on HNTs-M-chitosan (1%); (**B**) temperature stabilities of free and immobilized laccase; (**C**) time-duration stabilities of free and immobilized laccase; and (**D**) pH stabilities of free and immobilized laccase.

Immobilized enzymes may display enhanced biocatalytic properties [3]. Hence, after immobilization, it is important to assess the biocatalytic properties of the immobilized enzyme. We analyzed the laccase loading pattern by varying initial laccase concentration, the thermal stability performance, the temporal stability, and the pH stability of our immobilized laccase nanocomposites (Figure 7). Figure 7A shows the laccase loading as the initial laccase concentration was increased from 0.2 to 1.2 mg/mL. The laccase loading sharply declined for initial laccase concentrations above 1 mg/mL. At the initial laccase concentration of 1 mg/mL, HNTs-M-chitosan (1%)-GTA exhibited 99.61 mg/g of laccase loading. This may be due to the saturation of sites on the HNTs-M-chitosan (1%)-GTA available for immobilization. A similar effect was observed for the loading of the enzyme naringinase on chitosan microspheres [41]. The thermal stability is shown in Figure 7B. The free laccase lost 28% of its initial activity after incubating at 60 °C for 6 h. However, HNTs-M-chitosan (1%)-GTA-*Laccase* only lost 15% of the initial activity. The obtained results confirm that the immobilization of laccase on the HNTs-M-chitosan (1%)-GTA nanocomposites significantly enhanced the thermal stability of laccase. The binding of the enzyme to a support provides mechanical support to prevent enzymatic denaturation at higher temperatures [5]. 

The storage stability of free laccase and HNTs-M-chitosan (1%)-GTA-*Laccase* is shown in Figure 7C. The free laccase activity decreased over time, resulting in 44% activity lost and after 30 days of incubation. However, HNTs-M-chitosan (1%)-GTA-*Laccase* displayed higher stability compared to the free laccase, losing only 30% of their initial activity after 30 days. Similar storage stability improvements for immobilized laccase compared to free laccase were previously reported [29]. The pH of the local environment affect desired application of the enzyme [30]. Hence, the pH stability of HNTs-M-chitosan (1%)-GTA-*Laccase* is very important. Figure 7D shows the pH stability of free laccase and HNTs-M-chitosan (1%)-GTA-*Laccase*. The free laccase exhibited very strong stability in the pH range of 3–5. The HNTs-M-chitosan (1%)-GTA-*Laccase* showed excellent catalytical performance at the pH range of 1–6. The HNTs-M-chitosan (1%)-GTA-*Laccase* exhibited stability across a broad pH range, significantly higher than that of free laccase, especially at pH of 1–2 and pH 6–9. This indicates that the HNTs-M-chitosan (1%)-GTA-*Laccase* exhibit excellent stability and biocatalytic properties across a wide pH range.

**Figure 8:**
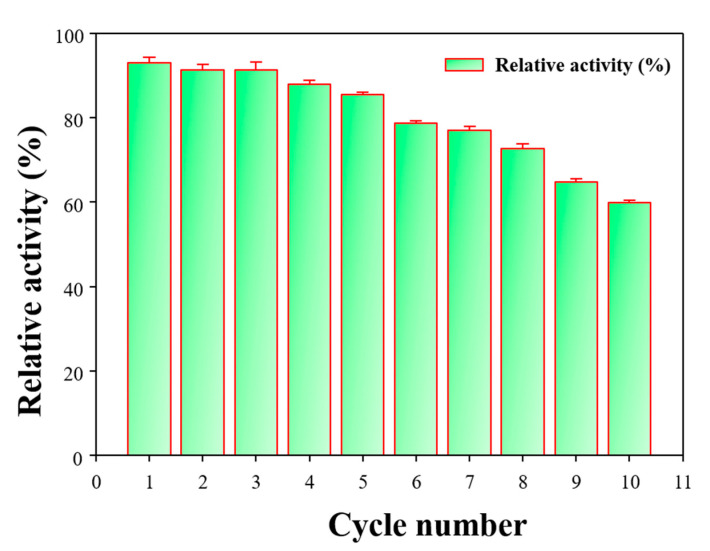
Repeated cycle bio-catalysis by HNTs-M-chitosan (1%)-GTA-*Laccase*.

Repeated use of the immobilized laccase is essential for its economic viability [42]. The repeated cycle study of the HNTs-M-chitosan (1%)-GTA-*Laccase* is shown in Figure 8. A total of 10 repeated cycles were performed. At the end of the 10th cycle, HNTs-M-chitosan (1%)-GTA-*Laccase* retained 60% of the initial activity. This result suggests exceptional reusability for the immobilized laccase, representing a significant improvement upon recently reported nano-supports for laccase immobilization [29,31,42,43]. Thus, the biocatalytic performance of the HNTs-M-chitosan (1%)-GTA-*Laccase* is excellent, resulting in a wide array of potential applications. 

### 3.5. Biocatalytic Degradation of SMX

**Figure 9:**
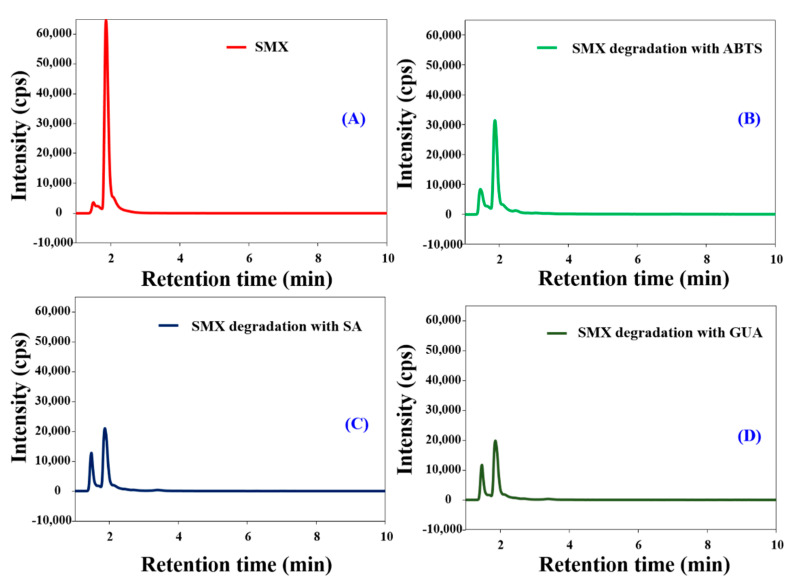
The high-performance liquid chromatography (HPLC) analysis of (**A**) sulfamethoxazole (SMX), (**B**) HNTs-M-chitosan (1%)-GTA-*Laccase* catalyzed SMX degradation with ABTS; (**C**) HNTs-M-chitosan (1%)-GTA-*Laccase* catalyzed SMX degradation with syringaldehyde (SA) and (**D**) HNTs-M-chitosan (1%)-GTA-*Laccase* catalyzed SMX degradation with guaiacol (GUA).

**Figure 10:**
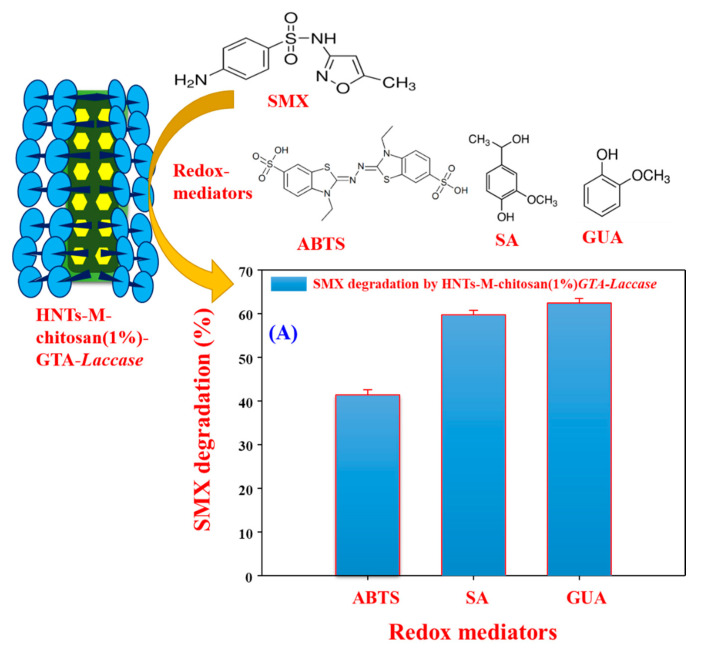
Schematic representation of redox-mediated SMX degradation by HNTs-M-chitosan (1%)-GTA-*Laccase* and (**A**) Data of SMX degradation by HNTs-M-chitosan (1%)-GTA-*Laccase* at pH 4.2 and 25 °C.

Pollution by pharmaceutical compounds poses serious environmental issues [42]; thus, their removal from water supplies is crucial. We used SMX as an example pharmaceutical compound pollutant. The HNTs-M-chitosan (1%)-GTA-*Laccase* does not degrade SMX in the absence of the redox mediator, because laccase requires a redox mediator compound to degrade SMX [10]. Immobilized laccase reduces the redox mediator compound, which then catalyzes the degradation of the target pollutant [22]. In this work, we used ABTS, SA, and GUA as redox mediators. The degradation of SMX by HNTs-M-chitosan (1%)-GTA-*Laccase* in the presence of ABTS, SA, and GUA was analyzed by HPLC (Figure 9A–D). Free SMX shows a clear and sharp peak at a retention time of 1.87 min (Figure 9A). The ABTS, SA, and GUA-mediated SMX degradation products exhibited two sharp peaks at 1.87 min and 1.45 min. However, the intensity of the 1.87 min peak decreased significantly compared to the standard SMX in presence of all the redox mediators. Hence, the split in standard SMX peak at 1.87 min and significant decrease in its intensity confirms the redox mediated breakdown of SMX by HNTs-M-chitosan (1%)-GTA-*Laccase*. Among all redox mediators, the GUA exhibited the highest decrease in the intensity of the 1.87 min peak of standard SMX. Thus, GUA found to be the best redox mediator than the ABTS and SA. The result is consistent with the previous report [10]. This might be due to the small molecular nature and easy way oxidation by laccase. The oxidized GUA by immobilized laccase immediately gets reduced by causing oxidative degradation of the SMX. This might accelerates the SMX degradation in presence of the GUA. The schematic of immobilized laccase, structures of SMX, ABTS, SA, and GUA and degradation percentage of SMX by redox mediators are shown in Figure 10. 

**Figure 11:**
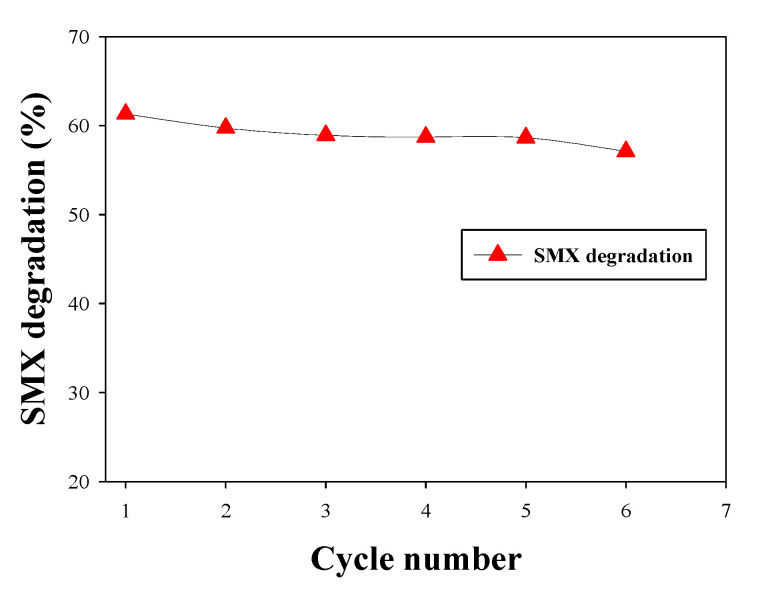
Cyclical degradation of SMX by HNTs-M-chitosan (1%)-GTA-*Laccase* in the presence of the redox mediator GUA, pH 4.2 at 25 °C.

The HNTs-M-chitosan (1%)-GTA-*Laccase* degraded 41%, 59%, and 62% of the SMX with ABTS, SA, and GUA, respectively (Figure 10). Cost effective and efficient technologies for environmental pollutants removal are highly important [44]. Hence, GUA is known to be green, valuable, economical, and beneficial mediator for laccase-mediated bioremediation applications [45]. Further, we performed six cycles of repeated SMX degradation (Figure 11), suggesting significant potential for the use of HNTs-M-chitosan (1%)-GTA-*Laccase* in a continuous process. The SMX degradation was observed to be 61.32, 59.73, 58.52, 58.72, and 58.65 in first five cycles of the repeated degradation. The HNTs-M-chitosan (1%)-GTA-*Laccase* degraded 57.10% of the SMX in the sixth cycle (Figure 11). This designates the strong potential of immobilized laccase. The small decrease of 4% was observed in six cycles. This might be due to the build-up of the degraded product. This result suggests that HNTs-M-chitosan (1%)-GTA-*Laccase* has significant potential for use in the biocatalytic degradation of pollutants, and can be applied in environmental decontamination processes. 

## 4. Conclusions

In-conclusion, we investigated the optimum loading of chitosan on the HNTs-M surface for efficacious laccase biocatalysis, and the degradation of the pharmaceutical compound SMX from water. Characterization by SEM, TEM, XRD, TGA, and VSM confirmed the successful synthesis of nano-support HNTs-M-chitosan (1%). HNTs-M-chitosan (1%)-GTA-*Laccase* displayed efficacious potential of redox mediated degradation of SMX. Thus, the HNTs-M-chitosan (1%)-GTA-*Laccase* may be an ideal system for the degradation of environmental pollutants. The developed nano-supports are economically cheap and applicable for encountering the problem of pharmaceutical compounds degradation. Additionally, the developed nano-support may be used to immobilize other enzymes to enhance their biocatalytic properties for a wide range of applications. 

## Figures and Tables

**Table 1:** The laccase loading capacity (mg/g) of recently reported nano-supports.

Material Name	Laccase Loading Capacity (mg/g)	Reference
MACS-NIL-Cu-Laccase	47	[28]
LA-Au/PDA@SiO2-MEPCM	50	[29]
Fe_3_O_4_@Chitosan	32	[30]
A-M-HNTs	84	[10]
M-PW-αCF-CTA	73	[24]
M-HNTs–CTA	92	[22]
Sepharose-linked antibody	33	[31]
Chitosan microspheres	8	[32]
PD-GMA-Ca@ABTS beads	8	[33]
Fe_3_O_4_-NIL-DAS@lac	60	[34]
HNTs-M-chitosan (1%)	100	This study

## References

[1] Su J., Fu J.J., Wang Q., Silva C., Cavaco-Paulo A. (2018). Laccase: A green catalyst for the biosynthesis of poly-phenols. Crit. Rev. Biotechnol..

[2] Bilal M., Zhao Y., Noreen S., Shah S.Z.H., Bharagava R.N., Iqbal H.M.N. (2019). Modifying bio-catalytic properties of enzymes for efficient biocatalysis: A review from immobilization strategies viewpoint. Biocatal. Biotransform..

[3] Fernandez-Lafuente R. (2019). Editorial for special issue: Enzyme immobilization and its applications. Molecules.

[4] Santos S., Puna J., Gomes J. (2020). A review on bio-based catalysts (immobilized enzymes) used for biodiesel production. Energies.

[5] Zhou W., Zhang W., Cai Y. (2020). Laccase Immobilization for Water Purification: A Comprehensive Review. Chem. Eng. J..

[6] Pathak N., Tran V.H., Merenda A., Johir 1 M.A.H., Phuntsho S., Shon H. (2020). Removal of organic micro-pollutants by conventional membrane bioreactors and high-retention membrane bioreactors. Appl. Sci..

[7] Zhang J., Zhao W., Li Z., Lu G., Zhu M. (2021). Visible-light-assisted peroxymonosulfate activation over Fe(II)/V(IV) self-doped FeVO4 nanobelts with enhanced sulfamethoxazole degradation: Performance and mechanism. Chem. Eng. J..

[8] Pandey G., Munguambe D.M., Tharmavaram M., Rawtani D., Agrawal Y.K. (2017). Manuscript title: Halloysite nanotubes-An efficient ‘nano-support’ for the immobilization of α-amylase. Appl. Clay Sci..

[9] Danyliuk N., Tomaszewska J., Tatarchuk T. (2020). Halloysite nanotubes and halloysite-based composites for environmental and biomedical applications. J. Mol. Liq..

[10] Kadam A.A., Jang J., Lee D.S. (2017). Supermagnetically Tuned Halloysite Nanotubes Functionalized with Aminosilane for Covalent Laccase Immobilization. ACS Appl. Mater. Interfaces.

[11] Lisuzzo L., Cavallaro G., Milioto S., Lazzara G. (2020). Halloysite Nanotubes Coated by Chitosan for the Controlled Release of Khellin. Polymers.

[12] Wang H., He J., Song L., Zhang Y., Xu M., Huang Z., Jin L., Ba X., Li Y., You L. (2020). Etching of halloysite nanotubes hollow imprinted materials as adsorbent for extracting of Zearalenone from grain samples. Microchem. J..

[13] Gómez L., Hueso J.L., Ortega-Liébana M.C., Santamaría J., Cronin S.B. (2014). Evaluation of gold-decorated halloysite nanotubes as plasmonic photocatalysts. Catal. Commun..

[14] Wu Y., Zhang Y., Ju J., Yan H., Huang X., Tan Y. (2019). Advances in Halloysite nanotubes-polysaccharide nanocomposite preparation and applications. Polymers.

[15] Tao Q., Bi J., Huang X., Wei R., Wang T., Zhou Y. (2021). Chemosphere Fabrication, application, optimization and working mechanism of Fe_2_O_3_ and its composites for contaminants elimination from wastewater. Chemosphere.

[16] Min K., Jee S.C., Sung J.S., Kadam A.A. (2018). Anti-proliferative applications of laccase immobilized on super-magnetic chitosan-functionalized halloysite nanotubes. Int. J. Biol. Macromol..

[17] Rojas-Lema S., Terol J., Fages E., Balart R., Quiles-Carrillo L., Prieto C., Torres-Giner S. (2020). Microencapsulation of Copper(II) Sulfate in Ionically Cross-Linked Chitosan by Spray Drying for the Development of Irreversible Moisture Indicators in Paper Packaging. Polymers.

[18] Lisuzzo L., Cavallaro G., Milioto S., Lazzara G. (2019). Layered composite based on halloysite and natural polymers: A carrier for the pH controlled release of drugs. New J. Chem..

[19] Lisuzzo L., Cavallaro G., Milioto S., Lazzara G. (2019). Core/Shell Gel Beads with Embedded Halloysite Nanotubes for Controlled Drug Release. Coatings.

[20] Bertolinoa V., Cavallaroa G., Miliotoa S., Lazzaraa G. (2020). Polysaccharides/Halloysite nanotubes for smart bionanocomposite materials. Carbohydr. Polym..

[21] Zabermawi N.M., Arif M., Elsaber G., Khafaga A.F., El-hakim Y.M.A. (2020). Antimicrobial and antioxidant properties of chitosan and its derivatives and their applications: A review. Int. J. Biol. Macromol..

[22] Kadam A.A., Jang J., Jee S.C., Sung J.S., Lee D.S. (2018). Chitosan-functionalized supermagnetic halloysite nanotubes for covalent laccase immobilization. Carbohydr. Polym..

[23] Piontek K., Antorini M., Choinowski T. (2002). Crystal structure of a laccase from the fungus Trametes versicolor at 1.90-A resolution containing a full complement of coppers. J. Biol. Chem..

[24] Ghodake G.S., Yang J., Shinde S.S., Mistry B.M., Kim D.Y., Sung J.S., Kadam A.A. (2018). Paper waste extracted α-cellulose fibers super-magnetized and chitosan-functionalized for covalent laccase immobilization. Bioresour. Technol..

[25] Bradford M.M. (1976). A rapid and sensitive method for the quantitation of microgram quantities of protein utilizing the principle of protein-dye binding. Anal. Biochem..

[26] Migneault I., Dartiguenave C. (2016). Glutaraldehyde: Behavior in aqueous solution, reaction with proteins, and application to enzyme crosslinking. J. Mater. Chem. A.

[27] Li X., Ouyang J., Yang H., Chang S. (2016). Chitosan modified halloysite nanotubes as emerging porous microspheres for drug carrier. Appl. Clay Sci..

[28] Qiu X., Wang S., Miao S., Suo H., Xu H., Hu Y. (2021). Co-immobilization of laccase and ABTS onto amino-functionalized ionic liquid-modified magnetic chitosan nanoparticles for pollutants removal. J. Hazard. Mater..

[29] Cao P., Liu H., Wu D., Wang X. (2020). Immobilization of laccase on phase-change microcapsules as self- thermoregulatory enzyme carrier for biocatalytic enhancement. Chem. Eng. J..

[30] Zhang K., Yang W., Liu Y., Zhang K., Chen Y., Yin X. (2020). Laccase immobilized on chitosan-coated Fe_3_O_4_ nanoparticles as reusable biocatalyst for degradation of chlorophenol. J. Mol. Struct..

[31] Zofair S.F.F., Arsalan A., Khan M.A., Alhumaydhi F.A., Younus H. (2020). Immobilization of laccase on Sepharose-linked antibody support for decolourization of phenol red. Int. J. Biol. Macromol..

[32] Aricov L., Leonties A.R., Gîfu I.C., Preda D., Raducan A., Anghel D.F. (2020). Enhancement of laccase immobilization onto wet chitosan microspheres using an iterative protocol and its potential to remove micropollutants. J. Environ. Manag..

[33] Xue P., Liu X., Gu Y., Zhang W., Ma L., Li R. (2020). Laccase-mediator system assembling co-immobilized onto functionalized calcium alginate beads and its high-efficiency catalytic degradation for acridine. Colloids Surf. B Biointerfaces.

[34] Qiu X., Wang Y., Xue Y., Li W., Hu Y. (2020). Laccase immobilized on magnetic nanoparticles modified by amino-functionalized ionic liquid via dialdehyde starch for phenolic compounds biodegradation. Chem. Eng. J..

[35] Katana B., Rouster P., Varga G., Muráth S., Glinel K., Jonas A.M., Szilagyi I. (2020). Self-Assembly of Protamine Biomacromolecule on Halloysite Nanotubes for Immobilization of Superoxide Dismutase Enzyme. ACS Appl. Bio Mater..

[36] Rouster P., Dondelinger M., Galleni M., Nysten B., Jonas A.M., Glinel K. (2019). Layer-by-layer assembly of enzyme-loaded halloysite nanotubes for the fabrication of highly active coatings. Colloids Surf. B Biointerfaces.

[37] Lazzara G., Cavallaro G., Panchal A., Fakhrullin R., Stavitskaya A., Vinokurov V., Lvov Y. (2018). An assembly of organic-inorganic composites using halloysite clay nanotubes. Curr. Opin. Colloid Interface Sci..

[38] Li N., Huang G.W., Shen X.J., Xiao H.M., Fu S.Y. (2013). Controllable fabrication and magnetic-field assisted alignment of Fe_3_O_4_-coated Ag nanowires via a facile co-precipitation method. J. Mater. Chem. C.

[39] Li X., Liu H., Deng Z., Chen W., Li T., Zhang Y., Zhang Z., He Y., Tan Z., Zhong S. (2020). PEGylated thermo-sensitive bionic magnetic core-shell structure molecularly imprinted polymers based on halloysite nanotubes for specific adsorption and separation of bovine serum albumin. Polymers.

[40] Zhong B., Jia Z., Hu D., Luo Y., Guo B., Jia D. (2016). Surface modification of halloysite nanotubes by vulcanization accelerator and properties of styrene-butadiene rubber nanocomposites with modified halloysite nanotubes. Appl. Surf. Sci..

[41] Bodakowska-Boczniewicz J., Garncarek Z. (2019). Immobilization of naringinase from penicillium decumbens on chitosan microspheres for debittering grapefruit juice. Molecules.

[42] Masjoudi M., Golgoli M., Nejad Z.G., Sadeghzadeh S., Borghei S.M. (2020). Pharmaceuticals Removal by Immobilized Laccase on Polyvinylidene Fluoride Nanocomposite with Multi-Walled Carbon Nanotubes. Chemosphere.

[43] Gou Z., Ma N.L., Zhang W., Lei Z., Su Y., Sun C., Wang G., Chen H., Zhang S., Chen G. (2020). Innovative hydrolysis of corn stover biowaste by modified magnetite laccase immobilized nanoparticles. Environ. Res..

[44] Lade H., Kadam A., Paul D., Govindwar S. (2015). A Low-Cost Wheat Bran Medium for Biodegradation of the Benzidine-Based Carcinogenic Dye Trypan Blue Using a Microbial Consortium. Int. J. Environ. Res. Public Health.

[45] Wong K.S., Cheung M.K., Au C.H., Kwan H.S. (2013). A Novel Lentinula edodes Laccase and Its Comparative Enzymology Suggest Guaiacol-Based Laccase Engineering for Bioremediation. PLoS ONE.

